# Circular RNA-Centered Regulatory Networks in the Physiopathology of Cardiovascular Diseases

**DOI:** 10.3390/ijms21020456

**Published:** 2020-01-10

**Authors:** André F. Gabriel, Marina C. Costa, Francisco J. Enguita

**Affiliations:** 1 Instituto de Medicina, Molecular João Lobo Antunes, Faculdade de Medicina, Universidade de Lisboa, Av. Prof. Egas Moniz, 1649-028 Lisboa, Portugal; andre.gabriel@medicina.ulisboa.pt (A.F.G.); marinacosta@medicina.ulisboa.pt (M.C.C.); 2 Cardiomics Unit, Centro Cardiovascular da Universidade de Lisboa, Av. Prof. Egas Moniz, 1649-028 Lisboa, Portugal; 3 Faculdade de Medicina, Universidade de Lisboa, Av. Prof. Egas Moniz, 1649-028 Lisboa, Portugal

**Keywords:** non-coding RNA, circRNAs, miRNAs, cardiovascular diseases, RNA regulatory networks

## Abstract

Non-coding regulatory RNAs are generated as a core output of the eukaryotic genomes, being essential players in cell biology. At the organism level, they are key functional actors in those tissues and organs with limited proliferation capabilities such as the heart. The role of regulatory networks mediated by non-coding RNAs in the pathophysiology of cardiovascular conditions is starting to be unveiled. However, a deeper knowledge of the functional interactions among the diverse non-coding RNA families and their phenotypic consequences is required. This review presents the current knowledge about the functional crosstalk between circRNAs and other biomolecules in the framework of the cardiovascular diseases.

## 1. Introduction

In eukaryotic cells, circular RNAs (circRNAs) are covalently closed RNA molecules generated by non-canonical back-splicing of coding and non-coding transcriptional units [1,2]. Besides to the known viral genomes constituted by covalently closed RNA, the first evidence of the existence of non-genomic functional circular RNA molecules in eukaryotic cells was described in the 1970s by the discovery and characterization of plant viroids and virusoids, infectious circular RNAs that are replicated by the cellular transcriptional machinery and can be transferred between plant cells [3,4,5]. Eukaryotic genomes were later identified as an internal source of circRNAs, being initially considered as bystander products of incorrect splicing events [6,7]. Nowadays, the availability of high-throughput RNA sequencing methods (RNAseq) together with specialized bioinformatics algorithms devoted to circRNA analysis has enabled the identification of thousands of circRNAs along with their cell and tissue-specific expression patterns [8,9].

**Figure 1:**
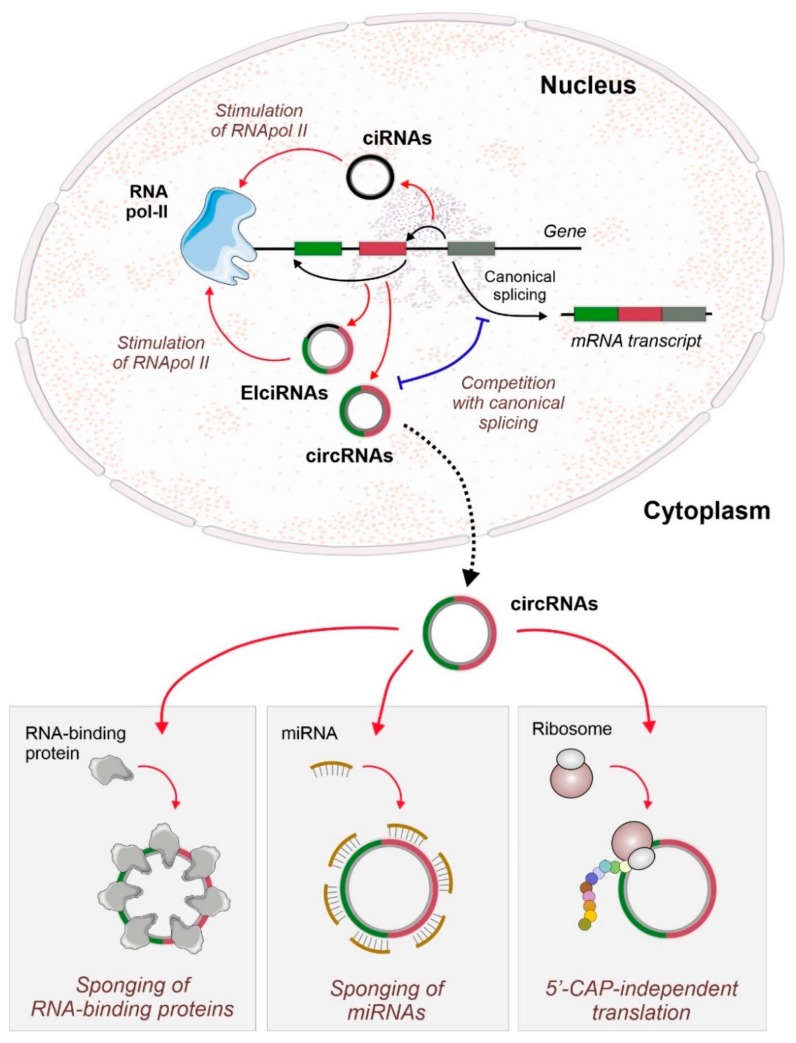
Biogenesis and function of circular RNAs (circRNAs). At the nucleus, back-splicing events competing with canonical splicing could generate several families of different circRNAs. Intron-containing circRNAs (circular-intronic RNAs, ciRNAs, and exonic-intronic circular RNAs, EIcircRNAs) can stimulate the transcription of their hosting gene by direct interaction with RNA polymerase II. At the cytoplasm, circRNAs functions include their action as sponges for RNA-binding proteins or microRNAs (miRNAs, and also as scaffolds of high-range complexes. Some circRNAs can also be translated in a 5’-CAP-independent manner to generate small or micro-peptides that can have regulatory functions.

CircRNAs biogenesis proceeds following a unique mechanism among non-coding RNAs (ncRNAs) since they are generated as by-products during the post-transcriptional processing of other RNA species (coding or non-coding). Jeck and coworkers proposed the term back-splicing to describe the pre-mRNA process of circularization (Figure 1) and covalent joining of the upstream 5’-splice acceptor to the downstream 3’-splice donor by phosphodiester bonds to generate a covalently closed RNA [10]. Two main models are suggested to explain circRNA formation: the lariat-driven circularization and the intron-pairing-driven circularization. In the lariat-driven model, circRNAs are originated from lariat intermediates that can contain both exons and introns and are formed during exon-skipping. In the intron-pairing circularization, there is a hybridization of introns flanking the back-spliced exons. The main players in this model are cis-acting elements (short non-coding regulatory sequences, as Alu repeats) located in the flanking introns that promote intron hybridization and facilitate exon circularization [10,11]. Additionally, RNA-binding proteins (RBPs) were also shown to be involved in circRNA biogenesis by acting as trans-acting factors that recognize and bind to specific motifs located in the introns flanking the back-spliced exons. By dimerization or protein–protein interaction, the RBPs are able to bridge two exon-bordering introns together, leading to RNA circularization. For instance, muscleblind protein (MBL) and quaking protein (QKI) are two *trans*-acting factors that regulate the formation of several circRNAs [12,13]. Regardless of the biogenesis model, circRNA isoforms can be originated from the same locus through alternative circularization [14]. Once the circular structure is formed, circRNAs become highly resistant to nuclease action, such as RNAse R [15,16], which means that their half-life is usually higher than other linear RNA molecules [17].

CircRNAs can be classified in three major groups: exonic circRNA (EciRNA or ecircRNA) generated by the circularization of one or more exons [10], intronic circRNA (ciRNA) originated solely from lariat-excised introns [18], and exon- and intron-containing circRNA (EIciRNA) [19]. CiRNAs and EIciRNAs are mainly found in the nucleus and are characterized as interaction partners of RNA-polymerase II, being present in the spliceosome, and even taking part in alternative splicing [19,20]. Increasing experimental pieces of evidence showed circRNAs as emerging regulatory players participating in the modulation of gene expression by sponging microRNAs (miRNAs) [2,16,21], by interacting with RBPs [12,13], and by regulating splicing and gene expression (Figure 1) [12,22]. 

CircRNAs, together with other ncRNA species such as miRNAs and lncRNAs, are pivotal players in the regulation of cardiovascular development and pathophysiology [23,24,25]. CircRNA-centered regulatory networks constitute a new regulatory layer of gene expression that would deserve a more detailed characterization, especially in those tissues with limited proliferation and very specialized in function such as the heart and the cardiovascular system [26].

## 2. CircRNAs as Active Players in the Regulation of Cardiovascular Diseases

### 2.1. MiRNA Sponging by circRNAs

**Figure 2:**
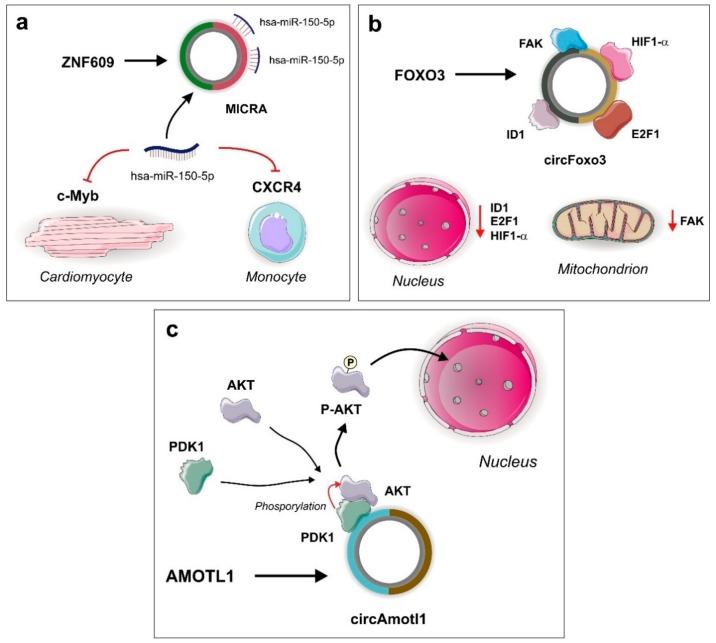
Selected circRNAs and their functional roles in cardiovascular diseases acting as cytoplasmic sponges and molecular scaffolds. (**a**) Myocardial infarction-associated circular RNA (MICRA) circRNA generated by back-splicing of the *ZNF609* gene acts as a molecular sponge of miR-150-5p. The sponging effect in cardiomyocytes is related with an increase of cellular activity by the up-regulation of its target, c-Myb [39,40]. (**b**) Cellular senesce related with cardiomyopathies in mouse heart is regulated by the protein-sponging function of circFoxo3 circRNA, which is able to capture the nuclear transcription factors ID1 and E2F1, and the antisenescence proteins HIF1-α and FAK, decreasing their levels and inducing cellular stress [41,42]. (**c**) CircAmotl1 suppress apoptosis events in cardiomyopathy-induced mouse models by facilitating the interaction between PDK1 kinase and its substrate AKT. Phosphorylated AKT originated from this scaffold-mediated interaction, is translocated to the nucleus and participates in the regulation of cell survival [43].

Regulatory crosstalk between non-coding RNA species was exemplified by the characterization of competing endogenous RNA (ceRNA) networks where an anchor RNA molecule was able to capture miRNAs by sequence complementarity, removing them from the medium and decreasing their regulatory effects over target mRNA transcripts. This phenomenon was firstly described in the differentiation of muscle, where the MD1 lncRNA acts as a molecular sponge of miR-133, activating muscle-specific gene expression [27], and also in tumor cells, where the mRNA of the tumor suppressor PTEN can behave like a sponge for several miRNAs, establishing a trans-regulatory network that affects gene expression in cancer [28]. CircRNAs could also contain miRNA regulatory or response elements (MREs), sequences that complementary bind to miRNA molecules, capturing them and inhibiting their functions in the cell (Figure 2) [2,21,29,30].

Several bioinformatics tools, such as CircInteractome, CircNet, Circ2Traits, and StarBase v2.0 have been of great benefit to predict miRNA binding sites in circRNAs [31,32,33,34]. Furthermore, the number of validated circRNA/miRNA interactions is increasing, being reported in practically every organ and tissue, not only in humans but also in other animal species [2,29,35,36,37]. Currently, miRNA sponging is the most well-characterized circRNA function. However, despite a large number of discovered cardiovascular expressed circRNAs [38], only a few have been reported to act as miRNA sponges (Table 1).

#### 2.1.1. Cdr1as (ciRS-7)

Cdr1as (ciRS-7) is a very well-known circRNA that acts as a miRNA sponge in numerous and different cell types such as cardiomyocytes, neural cells, and hepatic cells [2,29,44]. Cdr1as is up-regulated in myocardial infarction (MI) mice and it was described to act as a sponge for miR-7a in cardiomyocytes [29]. It was previously shown that miR-7a protects the heart from MI in hypoxic cardiomyocytes by negatively regulating the pro-apoptotic poly (ADP-ribose) polymerase (PARP) and the zinc finger Sp1 transcription factor, thus suppressing ischemia/reperfusion(I/R)-induced apoptosis [45]. Upon interaction with cdr1as, miR-7a becomes less abundant in the cardiomyocytes and PARP levels are higher, inducing apoptosis [29,45]. Thus, higher cellular levels of this circRNA are responsible for the aggravation of MI effects [29].

#### 2.1.2. MFACR

The mitochondrial fission and apoptosis-related circRNA (MFACR) is generated from the exon 5 of the SET and MYND domain-containing 4 (*Smyd4*) gene [46]. This circRNA is able to mediate mitochondrial fission and consequent activation of apoptotic events by acting as an miRNA sponge for miR-652-3p. In mammalian cells, mitochondrial fission and induced apoptosis are regulated by overexpression of mitochondrial protein 18 (MTP18) [47,48]. Similarly, Wang et al. described that MTP18 decrease led to an inhibition of mitochondrial fission and cardiomyocytes apoptosis [46]. In cultured cardiomyocytes, MTP18 expression is regulated by miR-652-3p and high levels of this miRNA in cells, contributing to the inhibition of mitochondrial fission and apoptosis. In vitro studies and bioinformatics analysis suggest that MFACR interacts with miR-652-3p in cardiomyocytes. In fact, MFACR contains 15 MREs for miR-652-3p and the overexpression of MFACR increased the protein level of MTP18 by sponging miR-652-3p. Moreover, inhibition of miR-652-3p would suppress the effect of MFACR knockdown on MTP18 [46].

#### 2.1.3. circNCX1

CircNCX1 is generated from the second exon of the sodium/calcium exchanger 1 (*ncx1*) gene and also mediates cardiomyocyte apoptotic events. Higher levels of reactive oxygen species (ROS) in the myocardium are related to increased levels of circNCX1 and may intensify the effects of I/R damage. In fact, circNCX1 can act as an miRNA sponge, harboring 8 binding sites for miR-133a-3p. This miRNA was previously described as cardioprotective in hypertrophic cardiomyopathy and heart failure by targeting the cell death-inducing p53-target protein 1 (CDIP1), an apoptotic transducer. Thus, circNCX1 sponge activity may suppress miR-133a-3p, leading to an increased number of apoptotic cardiomyocytes that intensify MI effects such as myocardial damage [49,50].

#### 2.1.4. HRCR

The heart-related circRNA (HRCR), generated from the murine *Pwwp2a* gene, was the first circRNA shown to play a cardioprotective role in hypertrophy and heart failure [37]. HRCR acts as an miRNA sponge, harboring 6 binding sites for miR-223 and down-regulating its activity. MiR-223 directly suppresses the mRNA transcript of the apoptosis repressor with CARD domain protein (ARC); however, decreased levels of ARC can induce hypertrophy due to cardiomyocyte apoptosis. By regulating miR-223, HRCR is responsible for a higher rate of ARC translation causing a decrease of apoptotic cardiomyocytes and subsequent cardiomyocyte hypertrophy. Ultimately, HRCR activity lessens the odds of heart failure [37].

#### 2.1.5. circS1c8a1

Another circRNA that plays a major role in cardiac hypertrophy is circSlc8a1. This circRNA was previously shown to be abundant in the human heart [38]. Lately, it has been confirmed that circSlc8a1 contains miRNA binding sites for miR-133a and, as mentioned before, this miRNA participates in cardioprotective processes. Inhibition of circSlc8a1 was proposed as a therapeutic approach in order to decrease the risk of dilated cardiomyopathy (DCM) and heart failure progression, resultant from pathological heart hypertrophy [51].

#### 2.1.6. circRNA_000203

CircRNA_000203 is a nine exon circRNA transcribed from the Myosin 9a (*Myo9a*) gene. It contains the exons 7 to 15 and respective flanking sequences of the introns 6 and 15. Despite its large size, this circRNA was shown to only harbor two MREs for miR-26b-5p, a suppressor of Col1a2 and CTGF expression [52]. Col1a2 is a peptide found in type I collagen and CTGF is an extracellular growth factor protein responsible for the regeneration of damaged fibrotic tissue. However, higher levels of these proteins may cause fibrosis. It is noteworthy that increased levels of Col3a1 and α-SMA proteins may also be found in cardiac fibroblasts [52]. Moreover, circRNA_000203 overexpression and its ability to sponge miR-26b-5p in fibroblasts were correlated to an increased risk of diabetic cardiomyopathy due to consequent Col1a2 and CTGF up-regulation [52].

#### 2.1.7. circRNA_010567

Another circRNA responsible for fibrotic tissue formation in diabetic cardiomyopathy conditions is circRNA_010567, which regulates the miR-141/TGF-β1 axis as an miR-141 sponge [53]. Down-regulation of this circRNA is responsible for a decrease in previously mentioned fibrotic protein levels such as Col1a2, Col3a1, and α-SMA, as well as TGF-β1, due to the suppression of circRNA_010567 sponge activity [53]. 

#### 2.1.8. circZNF609

The ZNF609 locus is found to generate two different circRNAs that have distinct roles in cardiovascular diseases, the myocardial infarction-associated circular RNA (MICRA) and circZNF609. MICRA is an 874 nt long circRNA originated from exon 1 of the *ZNF609* gene and proposed as a myocardial infarction biomarker [54]. Interestingly, MICRA is predicted to sponge miR-150-5p. It was shown in colon tissue that a similar circular transcript (circZNF609), derived from the same gene locus, regulates this miRNA [55]. Adding up the fact that miR-150 is up-regulated in left ventricular (LV) remodeling after acute myocardial infarction (AMI) [56], it is predicted that MICRA is able to regulate this miRNA; however, further investigation should be performed [54]. Moreover, circZNF609 was first identified to control skeletal myoblast proliferation in a knock-down-based study for the screening of circRNAs in Duchenne muscular dystrophy myoblasts, and it is originated from exon 2 of the *ZNF609* gene locus [57]. In this study, circZNF609 was overexpressed in endothelial cells from diabetes mellitus, hypertension, and coronary artery disease (CAD) patients, and was associated with increased angiogenesis [58]. On the other, circZNF609 was proposed as a potential miRNA sponge by interacting with miR-615, a negative regulator of the myocyte enhancer factor 2A (MEF2A), a transcription factor largely associated with CAD and MI [59]. In fact, in hypoxic and high glucose content conditions, circZNF609 was found to be up-regulated, leading to tube formation, migration of endothelial cells, and triggering programmed cell death. Conversely, the silencing of circZNF609 reduced the effects on endothelial damage [58,60].

#### 2.1.9. circ_000595 and circ_0010729

CircRNAs have been implicated in hypoxia-induced angiogenic events by sponging miRNAs. In an early study, hsa_circ_000595 RNA was associated with the increase of apoptosis in human aortic smooth muscle cells. Data showed that hsa_circ_000595 could target miR-19a and overexpression of the circRNA resulted in miR-19a down-regulation [61].

More recently, the circRNA hsa_circ_0010729 was described as a vascular cell apoptosis suppressor by inhibiting the activity of miR-186 [62]. In human umbilical vein endothelial cells, miR-186 appears to act as a pro-apoptotic factor down-regulating the activity of the hypoxia-inducible factor 1 alpha (HIF-1α), an anti-stress protein involved in angiogenesis under hypoxic conditions. CircRNA hsa_circ_0010729 could reverse the effects of hypoxia by regulating the vascular endothelial cell proliferation [62].

#### 2.1.10. circDLGAP4 and circHECTD1

Ischemic stroke events and other cerebral ischemic diseases are responsible for significant and serious impairment in the brain, with increased morbidity and mortality. Rising research on circRNAs has shown that these molecules are enriched in multiple organs and are highly expressed in the brain, suggesting that circRNAs may play an important role in regulating neurophysiological and pathophysiological processes [63].

Recent research provides evidence that the circular RNA circDLGAP4, originated from the back-splicing of exons 8 to 10 of the *DLGAP4* gene, may be implicated in the physiology of ischemic stroke [64]. CircDLGAP4 was found to be down-regulated in acute ischemic stroke patients and in a transient middle cerebral artery occlusion (tMCAO) mouse model. It was also demonstrated that circDLGAP4 could sponge miR-143, which can negatively modulate the expression of HECT domain E3 ubiquitin-protein ligase 1 (HECTD1). Noteworthy, it was reported that overexpression of circDLGAP4 could impact the reduction of neurological deficits and improve the damage of the blood–brain barrier, depicting the role of circDLGAP4 ischemic stroke [64].

The *HECTD1* locus is related to ischemic stroke development and regulation through the formation of the circular RNA circHECTD1 that contains exons 23 and 24 of the *HECTD1* gene [65]. CircHECTD1 was found to be associated with down-regulation of miR-142 expression in tMCAO, in human glioblastoma A172 cells treated with oxygen–glucose deprivation–reperfusion, and in acute ischemic stroke patients. This modulation led to astrocyte activation through the expression of autophagic proteins, leading to cerebral infarction. Pieces of evidence showed that TCDD-inducible poly (ADP-ribose) polymerase (TIPARP), an enzyme linked to ischemic stroke, could be a target for miR-142. In this setting, the down-regulation of circHECTD1 was correlated with lower levels of TIPARP, resulting in a lower risk for brain infarction [65].

### 2.2. Interaction of circRNAs with RNA-Binding Proteins

Recent studies started to show that circRNAs may also interact and modulate the cellular roles of many RNA-binding proteins (RBPs). These interactions are very complex, as different RBP/circRNA interactions could result in diverse regulatory events. However, two main circRNA/RBP interactions can be distinguished: RBP sponging and RBP scaffolding [2,41,66].

The RBP sponging function is very similar to the previously described miRNA sponging function. Essentially, some circRNAs contain RNA-protein binding sites that present high-affinity to certain RBPs that prevent their availability and consequent roles in the cell. CircRNA/protein interactions started to be described in 2014. *Drosophila melanogaster* muscleblind protein (MBL) was the first RBP found to strongly regulate circRNA synthesis from the *Mbl* locus and was also the first protein noticed to undergo RBP/circRNA sponging. Cellular MBL protein abundance is responsible for its own pre-mRNA back-splicing and circularization. This process prevents the formation of a linear transcript and consequent MBL protein formation and accumulation, in a negative feedback mechanism. More interesting is the fact that mature circMbl molecules contain many MBL binding sites with the ability to intake these proteins and balance their cellular amount [12].

Though many circRNAs act as RBP sponges, many other circRNAs own the ability to modulate protein function by acting as scaffolds that stabilize and enable protein–protein interactions. For instance, it was previously reported that circFoxo3, generated from the forkhead box-O 3 (*Foxo3*) gene, acts as a sponge for certain transcription factors and antisenescence proteins but also functions as a scaffold for p21 protein. circFoxo3 contains p21 binding sites and the interaction they establish facilitates the communication between p21 and Cdk2 protein, triggering the chain of events that prevent cell cycle progression [42].

Currently, only a limited number of circRNA/RBPs interactions have been described in the context of cardiovascular pathophysiology; however, a growing number of evidence has been reported through the last few years (Table 2).

#### 2.2.1. RBP Sponging by circRNAs

Transcription of the *Foxo3* gene is responsible for the generation of either linear or circRNA transcripts. The linear form is translated into Foxo3 protein, an important transcription factor involved in cell cycles, apoptosis, and autophagy processes. The levels of the circRNA circFoxo3 originated from the *Foxo3* gene are higher in the hearts of older mice and humans and are responsible for controlling cell senescence. An experiment using a model of doxorubicin-induced cardiomyopathy mice demonstrated that overexpression of circFoxo3 promotes heart cell senescence. Although circFoxo3 was not shown to interact with miRNAs, it contains canonical protein binding sites that can sponge the anti-stress transcription factors ID1 (DNA-binding protein inhibitor ID-1) and E2F1 (E2F transcription factor 1) and the antisenescence proteins HIF1α (hypoxia-inducible factor 1-alpha) and FAK (focal adhesion kinase 1). The interaction between these molecules results in the retention of these proteins in the cytoplasm. The decrease of ID1, E2F1, and HIF1α in the nucleus, and FAK in the mitochondria, is responsible for cellular stress and posterior senescence (Figure 2) [41].

The *CDKN2B-AS* gene, located in the chromosome 9p21.3, contains the information for the antisense (long) noncoding RNA in the INK4 locus, abbreviated as ANRIL. The same locus may produce a circular RNA form, circANRIL, which is associated with atherosclerotic vascular disease (ASVD). It was shown in HEK-293 cells that this circRNA interacts with the pescadillo homolog (PES1) protein. PES1 is a component of the PeBow complex and is responsible for the maturation and formation of the 60S ribosome subunit, being crucial in cell cycle progression. In atherosclerotic plaques, PES1 sponging by circANRIL has consequences in cell proliferation suppression and a consequent increase of cell apoptosis in proliferating cells. Thus, circANRIL is responsible for atheroprotection [67,68].

#### 2.2.2. circRNA Scaffolds

CircAmotl1 was shown to suppress apoptosis events in a doxorubicin-induced cardiomyopathy mouse model. In the cytoplasm of cardiomyocytes, circAmotl1 acts as a scaffold facilitating the interaction between the serine/threonine kinase AKT and the 3-phosphoinositide-dependent protein kinase 1 (PDK1). PDK1 phosphorylates AKT trigger the translocation of phosphorylated AKT (pAKT) into the nucleus. In the nucleus, pAKT participates in the regulation of cell survival processes. An experiment using antisense oligonucleotides for the AKT and PDK1 binding sites showed that the amount of dephosphorylated AKT in the cytoplasm was increased and therefore it could not be translocated into the nucleus. Furthermore, the use of ribonuclease A was enough to suppress the interaction between AKT and PDK1. These experiments suggested that circAmotl1 is a crucial “platform” for AKT activation and translocation into the nucleus and protects the heart from cardiomyopathy (Figure 2). Interestingly, human circAmotl1 only differs from rodent circAmotl1 due to the sequences present in their exon junctions [43].

#### 2.2.3. Other circRNA-Mediated Interactions

The titin (*TTN*) gene is the largest coding gene found in mammals. The RNA that results from its transcription may contain up to 363 exons [69]. Some of the resultant *TTN* isoforms are crucial in muscle contraction, regulation of calcium content in the muscle fiber, and cell cycle upkeep [69]. The *TTN* gene may also produce circular RNA forms [70]. However, while most RNA circles are single-exon, circTTN isoforms are very long and may contain up to 153 exons [38]. Like their linear counterparts, different circTTN secondary structures are yet to be described. circTTN 105–111 is a downstream effector of quaking isoform 5 (QKI5). QKI5 and other QKI isoforms suppress I/R-induced apoptosis and attenuate cellular senescence in doxorubicin-induced cardiomyocytes. As such, lower levels of circTTN 105-111 are responsible for a significant decrease in active QKI5 levels and cells are more prone to senescence. In short, although the circTTN 105–111 effect on QKI5 is still not very well understood, it plays a cardioprotective role alongside this RBP [71].

## 3. CircRNAs in Biofluids as Biomarkers of Cardiovascular Diseases

NcRNAs and other nucleic acids are actively secreted by cells, being present in all body biofluids and offering their potential use as disease biomarkers. The biochemical nature of ncRNAs offer better stability and flexible sample storage conditions, and increased sensitivity when compared with classical biomarkers [72]. Previous studies were successful to show the importance of circulating circRNAs as disease biomarkers and their potential uses as biological parameters that could be correlated with the onset, progression, or therapeutic response of cardiovascular diseases [54,73,74]. Detection and quantification of circRNAs in biofluids have been routinely performed either by hybridization microarrays for screening purposes, or by RT-qPCR with the use of divergent primers for individual quantification. Next-generation sequencing techniques are also starting to be applied but with a more limited extent when compared with microarrays or qPCR since they are dependent on the quality of the RNA samples and more expensive [75]. Due to their continuous covalently closed structure and high resistance to exoribonucleases, circRNAs are highly stable molecules in comparison with other RNAs that can be detected in biofluids such as miRNAs. In fact, some extracellular circRNA have been proposed and as possible disease biomarkers by using small cohorts of patients [8,54,75,76]. For instance, circulating hsa_circ_0001785 has been proposed as a detection biomarker for breast cancer [77] and hsa_circ_0124644 could be employed as a diagnosis biomarker for coronary artery disease (CAD) [76]. However, the clinical implementation of circulating RNAs, including circRNAs, as biomarkers in the medical practice of cardiovascular diseases is still in its infancy mainly due to technical limitations and lack of validation in extended patient cohorts.

### 3.1. MICRA

MICRA, previously mentioned in the Section 2.1.8 as being transcribed from the first exon of the *ZNF609* gene, was found in peripheral blood samples of acute myocardial infarction (AMI) patients. Two recent reports emphasized the possibility of using MICRA from blood as a biomarker to infer on left ventricular (LV) dysfunction and remodeling four months after AMI. MICRA’s blood levels were shown to be low in a cohort of MI patients when compared to healthy volunteers. Salgado-Somoza et al. also reported that lower levels of MICRA in the blood of patients at reperfusion time will present a reduced heart ejection fraction and that higher levels of blood MICRA are indicative of a mid-range or preserved ejection fraction [54,78].

### 3.2. circRNA_081881

Another circRNA that may be used to diagnose AMI is circRNA_081881. This molecule is down-regulated in the plasma of AMI patients alongside the peroxisome proliferator-activated receptor gamma (PPARγ) transcription factor. Deng et al. [73] performed siRNA-mediated knockdown assays for circRNA_081881 and found a decrease of blood PPARγ, suggesting that circRNA_081881 is directly related to PPARγ expression. In fact, the same group found seven MREs for miR-548, PPARγ being one of its targets [73].

## 4. Conclusions and Further Perspectives

The dynamic output of the human genome in the form of RNA transcripts contains a wide range of molecular species with different functions. The non-coding transcriptome constitutes by far the more diverse part of the genomic output, harboring regulatory ncRNAs that can control the information flow from the coding genome. The intricated functional relationships established between the different species belonging to the non-coding transcriptome are starting to be characterized in cell biology, but also in human disease. Among the ncRNAs, lncRNAs and circRNAs are very diversified in their functions due to their abilities to interact with different biomolecules including DNA, RNA, and proteins. In cardiovascular diseases, circRNAs have been characterized as important players in the control of the cellular levels of miRNAs and RNA-binding proteins, with different functional and pathological consequences ranging to direct involvement in pathology to protection from a cardiovascular condition.

Despite of the importance of circRNAs and the established regulatory networks, the availability of data about their roles in cardiovascular diseases is still limited due to the lack of knowledge about the rules governing their biogenesis from splicing events, the limitations of laboratory models, and also the lack of consensus within the scientific community in circRNA annotation and databases. A stronger effort is required to circumvent these limitations, since the central position of circRNAs in the regulatory events leading to some cardiovascular conditions may lead to potential new therapeutic and diagnostic strategies for better healthcare.

## Figures and Tables

**Table 1:** circRNAs acting as miRNAs sponges and modulating their function in cardiovascular diseases.

circRNA	circRNA Locus	MiRNA	MiRNA Target	Number of MRES ^1^	Disease	Effects of circRNA Up-Regulation
cdr1as	CDR1	miR-7a	PARP, SP1	63	Myocardial Infarction	Cardiomyocyte apoptosis and worsening of MI symptoms
MFACR	Smyd4	miR-652-3p	MTP18	15	Myocardial Infarction	Cardiomyocyte mitochondrial fission and apoptosis
circNCX1	Ncx1	miR-133a-3p	Cdip1	8	Myocardial Infarction	Cardiomyocyte apoptosis
HRCR	Pwwp2a	miR-223	ARC	6	Cardiac Hypertrophy and Heart Failure	Cardioprotective role by reduced apoptosis in hypertrophy cardiomyocytes
circSlc8a1	Slc8a1	miR-133a	Cdip1	17	Cardiac Hypertrophy and Heart Failure	Increased risk of dilated cardiomyopathy and heart failure progression
circRNA_000203	Myo9a	miR-26b-5p	Col1a2, CTGF	2	Cardiac Fibrosis in Diabetic Cardiomyopathy	Arrhythmia and heart failure due to fibrotic tissue
circRNA_010567	N/A ^2^	miR-141	TGF-β1	N/A ^2^	Cardiac Fibrosis in Diabetic Cardiomyopathy	Arrhythmia and heart failure due to fibrotic tissue
MICRA	ZNF609	miR-150	ADRB1, CRP	N/A ^2^	Coronary artery disease	Decreased LV disfunction risk
circZNF609	ZNF609	miR-615	MEF2A	1	Hypoxic angiogenesis and endothelial disorders	Worsening of endothelial damage
hsa_circ_000595	BTBD7	miR-19a	NF-κB, COX-2	N/A ^2^	Hypoxic angiogenesis and endothelial disorders	Aortic smooth muscle cell apoptosis. Aortic aneurism
hsa_circ_0010729	HSPG2	miR-186	HIF-1α	N/A ^2^	Hypoxic angiogenesis and endothelial disorders	Angiogenesis proliferation and apoptosis suppression
circDLGAP4	DLGAP4	miR-143	HECTD1	1	Stroke	Decreased neural deficits, decreased infarction area and mitigation of BBB damage
circHECTD1	HECTD1	miR-142	TIPARP	1	Stroke	Astrocyte activation and brain infarction

^1^ MRES: miRNA response elements. ^2^ N/A: not available.

**Table 2:** circRNAs involved in interactions with RNA-binding proteins and their roles in cardiovascular diseases.

circRNA	circRNA Locus	RBP ^1^	Protein-RNA Interaction	Disease	Effects of circRNA Up-Regulation
circFoxo 3	Foxo3	ID1, E2F1, HIF1α, FAK	RBP sponge	Cardiac senescence	Cardiomyocyte stress and senescence
circANRIL	CDKN2B-AS	PES1	RBP sponge	Atherosclerosis	Induced cell proliferation arrest and apoptosis Atheroprotection
circAmotl1	Amotl1	AKT, PDK1	Scaffold	Cardiomyopathy	Decreased cardiomyocyte senescence
circTTN 105-111	TTN	QKI5	Unknown interaction	Cardiac senescence	Decreased cardiomyocyte senescence

^1^ RBP: RNA-binding protein.
